# Microstructural Evolution at Micro/Meso-Scale in an Ultrafine-Grained Pure Aluminum Processed by Equal-Channel Angular Pressing with Subsequent Annealing Treatment

**DOI:** 10.3390/ma8115391

**Published:** 2015-11-04

**Authors:** Jie Xu, Jianwei Li, Xiaocheng Zhu, Guohua Fan, Debin Shan, Bin Guo

**Affiliations:** 1 Key Laboratory of Micro-systems and Micro-structures Manufacturing of Ministry of Education, Harbin Institute of Technology, Harbin 150080, China; xjhit@hit.edu.cn (J.X.); shandebin@hit.edu.cn (D.S.); 2 School of Materials Science and Engineering, Harbin Institute of Technology, Harbin 150001, China; 13B909069@hit.edu.cn (J.L.); zhuxc.hit@gmail.com (X.Z.); ghfan@hit.edu.cn (G.F.)

**Keywords:** microstructure, micro/meso-forming, ultrafine grains, ECAP, aluminum

## Abstract

Micro-forming with ultrafine-grained (UFG) materials is a promising direction for the fabrication of micro-electro-mechanical systems (MEMS) components due to the improved formability, good surface quality, and excellent mechanical properties it provides. In this paper, micro-compression tests were performed using UFG pure aluminum processed by equal-channel angular pressing (ECAP) with subsequent annealing treatment. Microstructural evolution was investigated by electron back-scattered diffraction (EBSD) and transmission electron microscopy (TEM). The results show that microstructural evolutions during compression tests at the micro/meso-scale in UFG pure Al are absolutely different from the coarse-grained (CG) materials. A lot of low-angle grain boundaries (LAGBs) and recrystallized fine grains are formed inside of the original large grains in CG pure aluminum after micro-compression. By contrast, ultrafine grains are kept with few sub-grain boundaries inside the grains in UFG pure aluminum, which are similar to the original microstructure before micro-compression. The surface roughness and coordinated deformation ability can be significantly improved with UFG pure aluminum, which demonstrates that the UFG materials have a strong potential application in micro/meso-forming.

## 1. Introduction

With the rapid development of micro-electro-mechanical systems (MEMS), a lot of miniaturized components are widely used for electronics, automobiles, aerospace, and biomedical devices [1,2]. Micro/meso-forming is becoming an important micro-manufacturing method having the potential to fabricate MEMS micro-parts due to its advantageous characteristics for mass production with controlled quality and low cost [3,4,5]. The research on micro/meso-forming technology was mainly initiated by M. Geiger [6] at the beginning of the 1990s and there is evidence that the presence of size effects may lead to a breakdown in these basic manufacturing characteristics when the feature size of the specimen dimensions are less than 1 mm, belonging to the micro/meso-scale. To explore size effects in micro/meso-forming, many attempts have been conducted, and Vollersen *et al.* [7] summarized size effects in the micro-forming of metallic components in 2011. Chan and Fu [8,9] systematically studied the effects of geometry and grain size on the micro/meso-scale deformation behavior. Keller *et al.* [10] studied the microstructural aspects of the grain and specimen size effects on the mechanical properties of high-purity nickel, and the results show the presence of three different mechanical behaviors: polycrystalline, multicrystalline, and quasi-single crystalline, depending on the thickness and the number of grains across the thickness. Ghassemali *et al.* [11] investigated the effect of cold-work on the Hall-Petch breakdown in copper during micro-compression, and suggested analytical validation of the interactive effects of the specimen grain-subgrain dimensions on the mechanical behavior in micro-forming. Wang *et al.* [12] investigated the size effects during a uniaxial compression test when the specimens had only a few grains across the specimen diameter. Zhang and Dong [13] studied the effects of micro-forming based on a crystal plasticity finite element method (FEM), including geometrically necessary dislocations. Ran *et al.* [14,15] proposed a hybrid model by considering the phase composition and distribution of material for the evaluation of the size effect on fracture surface morphology and fracture formation. Therefore, the grain size effect on micro/meso-scale deformation behavior becomes a decisive issue in micro-forming processes of metallic materials.

Hopefully there is a way to solve grain size effects in micro/meso-forming by applying ultrafine-grained (UFG) materials with sub-micrometer or even nano-scale grain sizes produced by severe plastic deformation (SPD) [16,17,18,19], because ultrafine grains can improve the formability, surface quality, and high mechanical properties of MEMS components [20,21,22]. However, as the grain size decreases to the submicron range, micro-deformation behavior changes from dislocation-dominated in large grains to grain boundary-dominated in small grain regimes. Yu *et al.* [23] studied deformation behavior in UFG pure aluminum processed by equal-channel angular pressing (ECAP) and post-annealed specimens at room temperature (RT), and the results show that different work hardening behavior were observed during a macro-compression test when the grain size increased from 0.35 μm to 45 μm. Le *et al.* [24] investigated the influence of grain size ranging from 0.5 μm to 5.2 μm on the deformation microstructure using compression tests at macro-scale in aluminum prepared by a spark plasma sintering method. The results demonstrate that there is a strong correlation between microstructure and grain size in the near-micrometer regime. Sabirov *et al.* [25] investigated the effect of strain rate on the micro-compression behavior of UFG pure aluminum and the results demonstrate that a lower strain rate causes the activation of micro shear banding. Wang and Shan [26] studied the effect of strain rate on the tensile behavior of UFG pure aluminum and the results show that the deformation mechanism at lower strain rates in UFG pure aluminum may be related to grain boundary sliding. Thus, it is believed that grain boundary processes, such as grain boundary sliding and grain rotation, are the main deformation mechanism in UFG materials. However, there is only limited information available on micro/meso-deformation behavior when the material grain size is reduced to the sub-micrometer level, although these problems and limitations are beginning to attract attention within the materials science community. At present, Xu *et al.* [27] studied the effect of grain size and specimen dimensions on the micro-forming of high-purity aluminum and found there is a transition from strain hardening to strain softening with decreasing grain size during micro-compression, but the transition grain size is dependent upon the size of the specimen. Okamoto *et al.* [28] investigated the specimen and grain size dependence of compression deformation behavior at the micro-scale in electrodeposited nanocrystalline copper with average grain sizes of 360, 100, and 34 nm. The results demonstrate that the deformation mechanisms for pillars with nanocrystaline grain size (34 nm) are different from those for pillars with submicron grain size (360 nm) from the surface microstructure of deformed micropillars. There are few studies on microstructural evolution at the micro/meso-scale in UFG pure materials, but it is very important to determine the micro/meso-deformation mechanism which is hitherto unclear during the micro-forming process.

In this paper, micro-compression tests with a specimen size of 2 mm in diameter were performed using UFG pure aluminum processed by ECAP with subsequent annealing treatment. Micro/meso-deformation behavior of the sample with different grain sizes was investigated, and microstructural evolution of compressive samples was analyzed by electron back-scattered diffraction (EBSD) and transmission electron microscopy (TEM). This study is an attempt to prove that UFG pure aluminum has a significant potential for micro-forming technology.

## 2. Experimental Materials and Procedures

**Figure 1:**
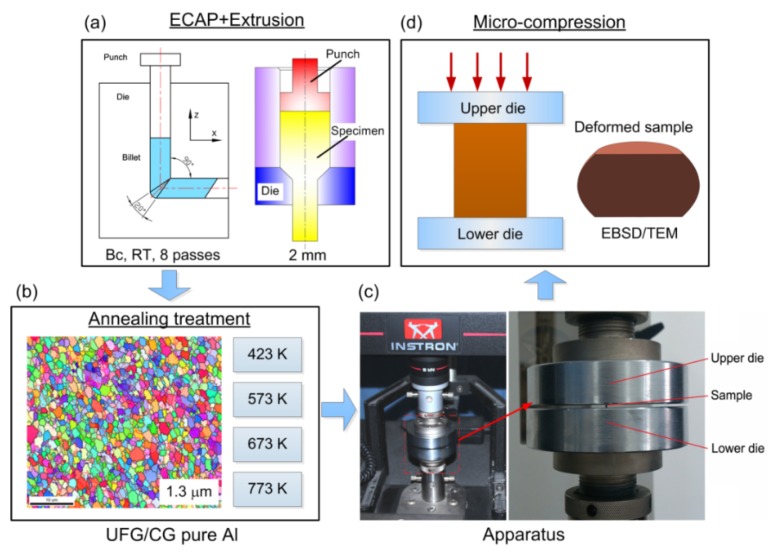
(**a**) Preparation of small specimen of UFG pure Al by ECAP and micro-extrusion; (**b**) Annealing treatment of pure Al; (**c**) Micro-compression apparatus; (**d**) Observation of microstructure of deformed sample by EBSD and TEM.

The experiments were conducted using a very high purity (99.999%) aluminum supplied in the form of drawn rods with diameters of 10 mm and lengths of ~70 mm. An annealing treatment was performed at a temperature of 773 K for 1 h by furnace cooling, and then the ECAP processing was conducted at RT using a die with an internal angle of 90° through eight passes using processing route B_c_ as shown in Figure 1a. A UFG pure Al was obtained with an average grain size of ~1.3 µm [29]. Following ECAP processing, micro-compression billets were prepared by micro-forward extrusion with a dimensional tool of 2 mm in diameter at room temperature. These extruded rods were cut and ground to micro-compression specimens with dimensions of 2 mm × 3 mm. It should be noted from Figure 1b that there is no change in grain size after micro-extrusion by comparison with the UFG pure Al processed by ECAP. Before micro-compression testing, the micro-compression specimens were annealed for 1 h at 423, 573, 673, and 773 K, respectively. The average grain sizes were calculated in the recent paper [25] as shown in Table 1. The average grain size increased from ~1.5 μm to ~150 μm with increasing annealing temperature from 423 K to 773 K. Following annealing treatment, all samples were electro-polished to smooth surfaces using a solution of 10% HClO_4_ and 90% C_2_H_5_OH with a DC voltage of 35 V at a temperature of 253 K. Micro-compression tests were conducted using an Instron testing machine (shown in Figure 1c,d) at room temperature with compression amounts of 30% and 50% at an initial strain rate of 1.0 × 10^−2^ s^−1^. In order to avoid any frictional size effect in micro-compression, no lubricant was used in the experiments [30].

**Table 1:** Grain size of pure Al processed by ECAP and extrusion with subsequent annealing treatment.

Conditions	After ECAP + Extrusion	423 K, 1 h	573 K, 1 h	673 K, 1 h	773 K, 1 h
Grain size (μm)	~1.3	~1.5	~4.0	~25	~150

After micro-compression testing, the surface morphologies of the compressed samples were examined using a Quanta 200FEG field emission scanning electron microscope (FESEM). Microstructural evolution after micro-compression was investigated by EBSD using the Quanta 200FEG FESEM with a working distance of ~13 mm and the data were analyzed with a TSL orientation imaging microscopy (OIM) software contained in the SEM. For EBSD measurements, the compressed cylinders were cut along the radial direction to obtain samples with a longitudinal plane for microstructure measurement as shown in Figure 1d. These longitudinal planes of the samples were ground on SiC papers up to 2000 grit and then electro-polished with the same polishing solution until a mirror-like surface was achieved. To establish the settings of the OIM analysis, the scanning step size was pre-determined according to the measured area and the anticipated grain size. Specifically, EBSD measurements were conducted over the area of 20 μm × 20 μm using a step size of 0.15 μm for the UFG samples, the area of 80 μm × 80 μm using a step size of 0.55 μm for the samples with grain size of ~4.0 μm, and the area of 300 μm × 300 μm using a step size of 2 μm for the samples for the coarse structures. The different misorientations between grains were determined with a minimum misorientation resolution of 2°. The misorientation angle distribution statistics were analyzed employing a critical misorientation angle of 15° to differentiate low-angle grain boundaries (LAGBs) from high-angle grain boundaries (HAGBs). Furthermore, transmission electron microscopy (TEM) measurements were also conducted on the cross-sectional plane of the compressed samples. Due to the small dimensions of the deformed samples, TEM specimens with a thickness of 50 nm were prepared with a focused ion beam (FIB) milling apparatus at an operating voltage of 30 kV in a two-beam SEM (HELIOS NanoLab 600i) system. Then, a field emission TEM (Tecnai G2 F30, FEI Instruments Co., Ltd., Hillsboro, OR, USA) was used for selected-area electron diffraction (SAED) analysis of microstructure observations at an operating voltage of 30 kV.

## 3. Experimental Results and Discussion

### 3.1. Flow Stress Behavior of Micro-Compression in Pure Al

**Figure 2:**
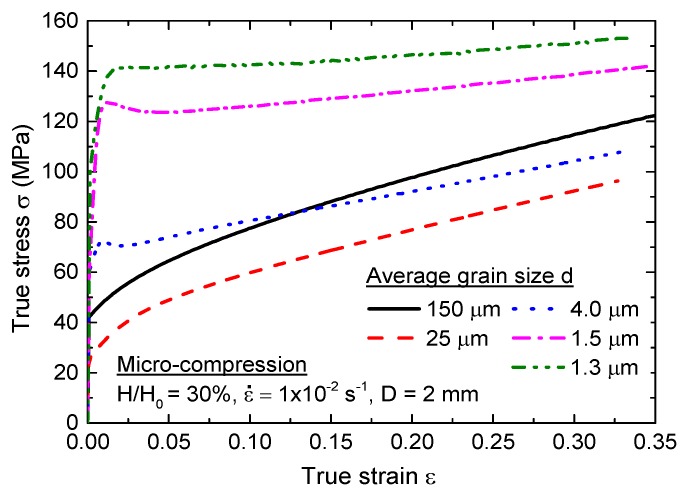
The curves of true stress and true strain after micro-compression testing with different grain size for a specimen size of 2 mm in diameter.

Figure 2 shows the plot of true stress *versus* true strain during micro-compression testing with different grain sizes for a specimen diameter of 2 mm at a strain rate of 1.0 × 10^−2^ s^−1^ and engineering compression strain of 30%. The results show that a UFG pure Al specimen without annealing treatment has the highest yield stress and flow stress, with flow stress and field stress decreasing with increasing grain size from ~1.3 µm to ~25 µm, consistent with the results of cold-drawn specimens [31]. The grain size effect on mechanical properties at the micro/meso-scale can be explained by the surface layer model proposed by Geiger [6]. There is an anomalous deformation behavior of the flow stress and yield stress in coarse-grained (CG) pure Al with a grain size of 150 µm, however, where both stresses are larger than that after micro-compression testing of specimens with a grain size of ~25 µm. Furthermore, it can also be observed that the micro-deformation behavior is transferred from typical work hardening for the sample in annealed condition to slight strain weakening in UFG pure Al after ECAP processing.

**Figure 3:**
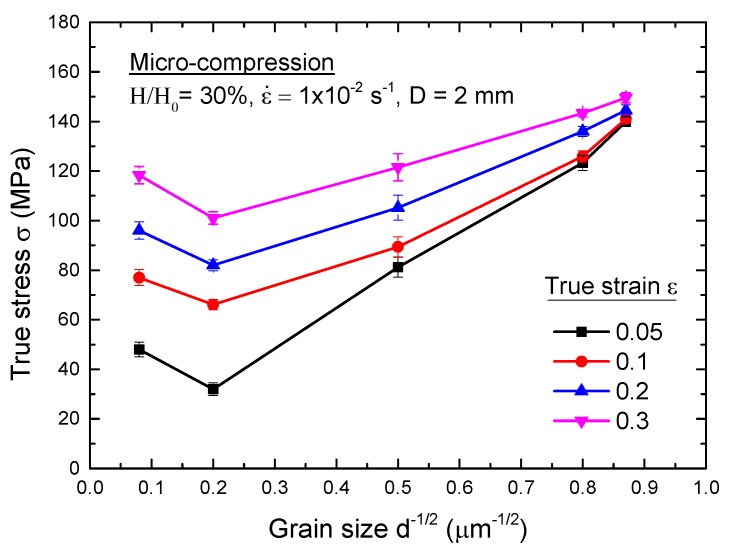
The Hall-Petch relationship after micro-compression testing with different grain sizes for a specimen size of 2 mm in diameter.

To check on the applicability of the Hall-Petch relationship, the values of true stress with standard error bars were plotted against d^−1/2^ at true strains of 0.05, 0.1, 0.2, and 0.3, as shown in Figure 3. These plots show that the true stress increases with decreasing grain size, which demonstrates that the Hall-Petch relationship is validated when d^−1/2^ > 0.2 µm^−1/2^. According to the Hall-Petch relation, the yield stress *σ* is related to the grain size *d* by the Equation:

(1) $$\sigma =\sigma_{0}+kd^{-1/2}$$

where $\sigma_{0}$ and k are constants. The Hall–Petch relation is verified by the experimental data for metals with grain size range from about 20 nm to hundreds of micrometers at the macro scale. However, there is an anomalous increase in Hall-Petch plot slope during micro-compression in CG pure Al with average grain size of ~150 μm. In this case, the contribution of the grain interior related to the density of dislocation accumulated in dislocation grain boundaries (or LAGBs). The Hall-Petch relation can be modified by Equation (2), proposed by N. Hansen [32]:

(2) $$\sigma_{f}=\sigma_{0}+\text{MαG}\sqrt{1.5b{\left(S_{V}\theta \right)}_{LAGB}}+k_{1}\left(\varepsilon \right)d_{HAGB}^{-1/2}$$

where $\sigma_{0}$ is the appropriate friction stress, *M* is the Taylor factor, α is a constant, *G* is the shear modulus, *b* is the Burgers vector, $S_{V}$ is the area of boundaries per unit volume and *θ* is the misorientation angle. Therefore, this anomalous increase in the Hall-Petch plot slope at thew micro/meso-scale in CG pure Al can be explained by the contributing effect of LAGBs, which will be verified by EBSD measurements.

### 3.2. Surface Topography after Micro-Compression in Pure Al

**Figure 4:**
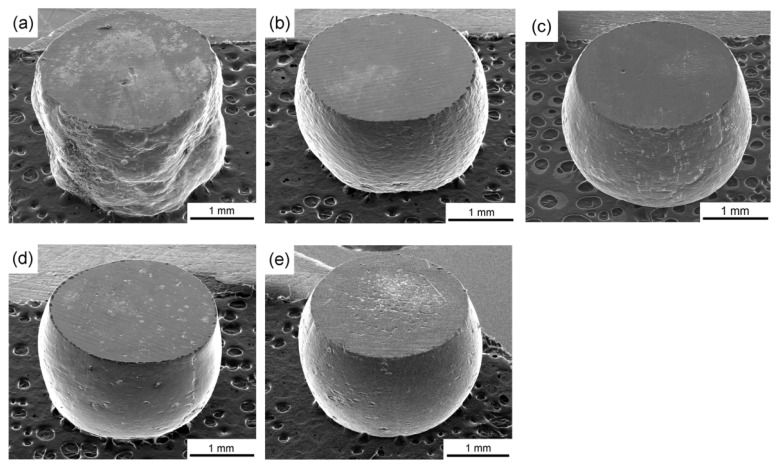
Surface topographies of the compressed sample with grain size of (**a**) ~150; (**b**) ~25; (**c**) ~4; (**d**) ~1.5; and (**e**) ~1.3 μm when compressed with a fixed specimen diameter 2 mm.

Figure 4 shows the surface topographies of the compressed specimens with average grain sizes of ~150, ~25, ~4.0, ~1.5, and ~1.3 μm when compressed with a fixed specimen diameter of 2 mm at a compression reduction of 50%. Inspection shows that the geometries of the compressed sample becomes irregular and the surface profile is not circular for the CG pure Al with a grain size of ~150 μm as shown in Figure 4a. There is an obvious transition from non-uniform deformation to uniform deformation by observing the degree of surface deformation after micro-compression testing with decreasing grain size as shown in Figure 4b–e. The non-uniform deformation can be improved significantly and the compressed specimens using cylindrical UFG pure Al with a smooth surface as shown in Figure 4d,e. These results demonstrate that an obvious surface-roughening effect occurs in micro-compression when the deformed specimen contains several grains, and this effect can be significantly improved with decreasing the grain size.

**Figure 5:**
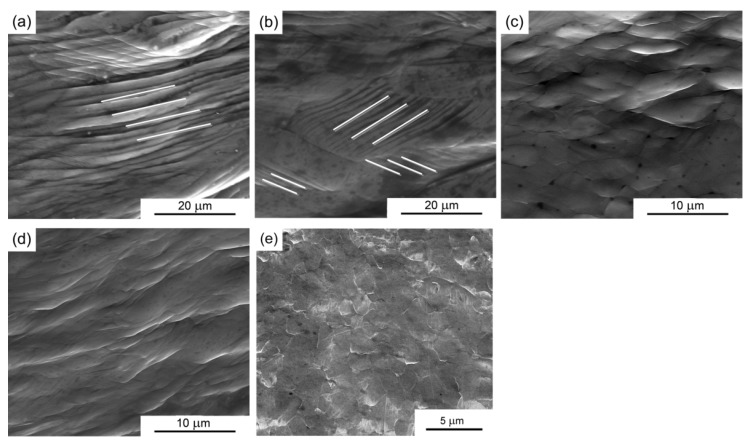
Microstructures on the surface of the compressed samples with grain size of (**a**) ~150; (**b**) ~25; (**c**) ~4; (**d**) ~1.5; and (**e**) ~1.3 μm when compressed fixed specimen diameter 2 mm.

The surface roughening phenomenon in micro/meso scale deformation affects the material formability, surface quality, and dimensional accuracy of the micro-parts and accelerates the die wear due to the increase of interfacial friction, which must be taken into account in micro-forming system design and micro-part quality control [3]. In order to investigate the effect of compatible deformation on surface roughening, the microstructures on the surface of the corresponding compressed samples in Figure 4 with average grain size of ~150, ~75, ~30, ~4, and ~1.5 µm were observed by SEM as shown in Figure 5. For the compressed samples with an average grain size of ~150, slip bands are clearly seen and are marked with white lines in Figure 5a. These slip banks run presumably to traces on one slip planes in the grains. For the compressed sample with a grain size of ~25 µm, deformation markings still can be found with a special direction in each grain, but the direction of the slip bands is different among the different grains, as shown in Figure 5b. These results indicate that the surface roughening effect in micro/meso-deformation is caused by the difficult compatible deformation between the neighboring coarse grains [33]. With decreasing grain size to less than ~4.0 μm, slip bands markings are hardly observed by SEM on the surface of compressed samples as shown in Figure 5c–e. Moreover, ultrafine grains and grain boundaries can be clearly observed, especially in the samples with UFG pure Al as shown in Figure 5e. Therefore, the significantly improved surface roughness after micro-compression tests in the UFG pure Al demonstrates that the UFG materials have a potential application in micro/meso-forming.

### 3.3. EBSD Evaluation of Microstructure after Micro-Compression in Pure Al

#### 3.3.1. Grain Size

**Figure 6:**
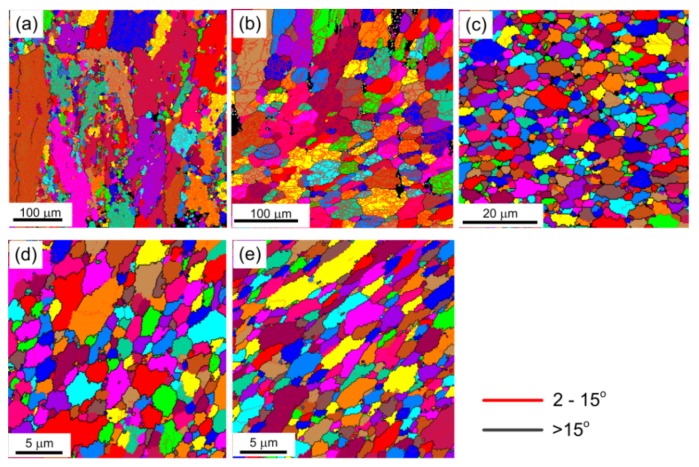
Microstructural varieties after micro-compression through compression amounts of 30% in pure Al with average grain sizes of (**a**) 150 µm; (**b**) 25 µm; (**c**) 4 µm; (**d**) 1.5 µm; and (**e**) 1.3 µm.

Figure 6 shows the OIM images of microstructural variety in the center of the deformed sample after micro-compression testing through compression amounts of 30% in pure aluminum with average grain sizes of ~150, ~25, ~4, ~1.5, and ~1.3 µm. Table 2 shows the corresponding results of fraction of LAGBs and HAGBs before and after micro-compression. For the sample of CG pure Al with a grain size of ~150 µm, there are many LAGBs formed inside of the coarse grains and some small recrystallized grains generated near the grain boundaries, as shown in Figure 6a. For the sample with a grain size of ~25 µm, the recrystallized grains decrease significantly, but there are still a lot of LAGBs in the large grains as shown in Figure 6b. Inspection of Table 2 shows that LAGBs increase evidently from 29.0% of initial undeformed sample to 83.2% after micro-compression for the CG pure Al with an average grain size of ~150 µm. The fraction of LAGBs for samples with an average grain size of ~25 µm also increases from 52.0% to 80.3% after micro-compression at the same conditions. These results demonstrate that the deformation mechanism at the micro/meso-scale in the CG pure Al is grain internal deformation with high density dislocations accumulation during micro-compression. These LAGBs evolutions also demonstrate the anomalous larger true stress and high Hall-Petch plot slope in CG pure Al with an average grain size of ~150 µm due to the increased contribution of LAGBs, according to the modified Hall-Petch relation in Equation (2). In further decreasing grain size from ~25 µm to ~4.0 µm, the fraction of LAGBs decreases obviously from 80.3% to 38.1% after micro-compression testing as shown in Figure 6b,c. The contribution of LAGBs on flow stress also can be neglected for the sample with a fine grain size of ~4.0 μm due to the small fraction of LAGBs. In addition, the microstructure has little change, including a slight reduction of grain size to ~2.0 µm and a small increase of the fraction of LAGBs from 31.7% in the original sample to 38.1% after micro-compression. For the UFG pure Al with a grain size of ~1.5 μm, it is found that there is no obvious change in microstructure and the ultrafine grains can be kept with an average grain size of ~1.5 µm after micro-compression for the corresponding UFG sample shown in Figure 6d. For the UFG sample with a grain size of ~1.3 µm after extrusion without any annealing treatment, the ultrafine microstructure remained but some grains were elongated due to the texture generated during forward extrusion as shown in Figure 6e. Moreover, there is a small increase in the fraction of HAGBs from 70.8% to 74.9% for the UFG pure Al with a grain size of ~1.3 µm, as shown in Table 2. This microstructural evolution demonstrates that the dominated deformation mechanism at the micro/meso-scale is dominated by compatible deformation by boundaries sliding and grain rotation during micro-compression in the UFG pure Al.

**Table 2:** Fraction of LAGBs and HAGBs before and after micro-compression.

Grain Size (µm)	Fraction of LAGBs (%)		Fraction of HAGBs (%)	
	Original Sample	After Compression	Original Sample	After Compression
~150	29.0	83.2	71.0	16.8
~25	52.0	80.3	48.0	19.7
~4.0	31.7	38.1	68.3	61.9
~1.5	28.2	31.5	71.8	68.5
~1.3	29.2	25.1	70.8	74.9

#### 3.3.2. Compression Amount

**Figure 7:**
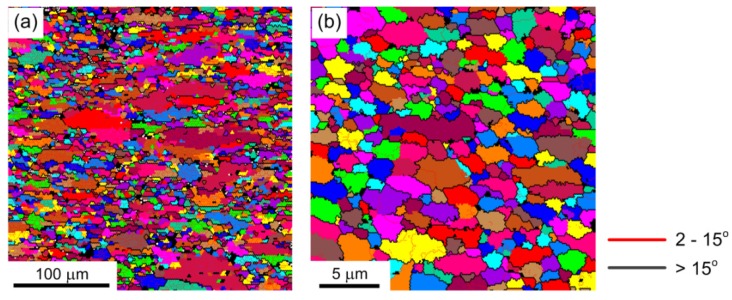
Microstructure in the center of the compressed sample through a compression amount of 50% in pure Al with average grain size of (**a**) ~25 µm and (**b**) ~1.5 µm.

**Figure 8:**
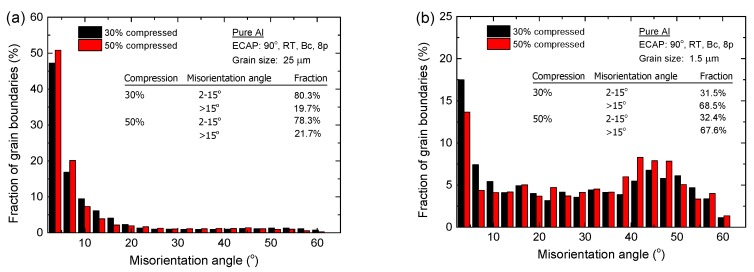
Histograms of the misorientation angles after micro-compression through different compression amounts in pure aluminum with an average grain size of (**a**) ~25 µm and (**b**) ~1.5 µm.

Figure 7 shows the microstructures in the center of the deformed samples after micro-compression through compression amounts of 50% in pure Al with average grain sizes of ~25 µm and ~1.5 µm. Figure 8 shows the histograms of the fraction of grain boundaries with misorientation angles after micro-compression through compression amounts of 30% and 50% in pure aluminum with average grain sizes of ~25 µm and ~1.5 µm. The results show that the microstructure of the sample with a grain size of ~25 µm after a micro-compression of 50% in Figure 7a is distinctly different from the microstructure after a micro-compression of 30% in Figure 6b. A lot of recrystallized fine grains are formed near the grain boundaries with increasing the amount of compression from 30% to 50%. In contrast, there are no obvious changes in microstructure remaining in the equiaxed grains for the UFG pure Al with a grain size of 1.5 µm, not only after micro-compression of 30% as shown in Figure 6d, but also after micro-compression of 50%, shown in Figure 7b. However, both the fractions of LAGBs and HAGBs have little change after micro-compression with increasing the amount of compression from 30% to 50%, not only in the CG pure Al with a grain size of ~25 µm, but for the UFG pure Al with a grain size of ~1.5 µm. These results demonstrate that the fraction of grain boundaries is not significantly affected by the amount of compression during micro-compression.

#### 3.3.3. Deformed Region

**Figure 9:**
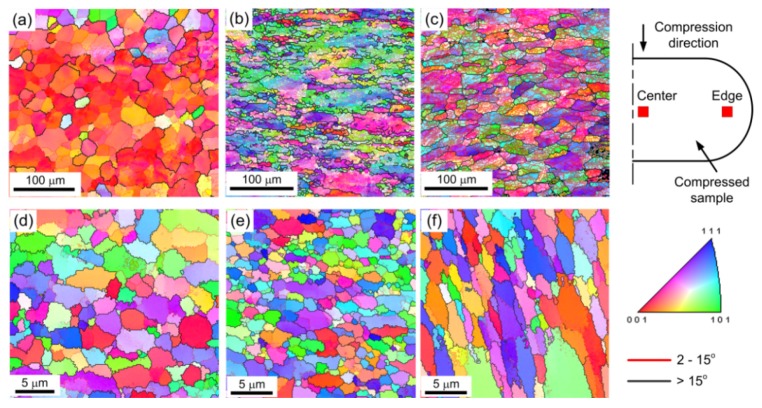
Microstructural evolution in different regions during micro-compression in pure Al: (**a**) microstructure of the initial undeformed sample, (**b**) in the center, and (**c**) in the edge after micro-compression of 50% in pure Al with a grain size of ~25 µm; (**d**) microstructure of initial undeformed sample, (**e**) in the center, and (**f**) in the edge after micro-compression of 50% in pure Al with a grain size of ~1.5 µm.

**Figure 10:**
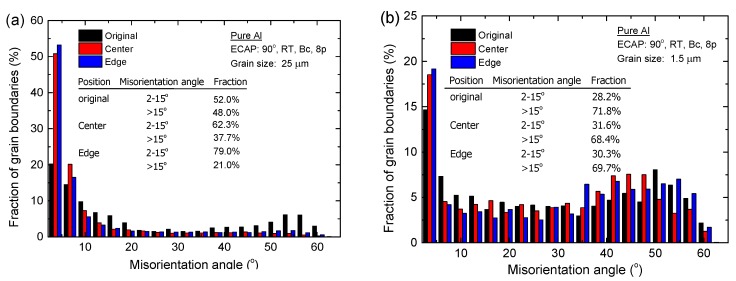
Comparison of the fraction of grain boundaries in different regions of compressed samples of pure Al with average grain sizes of (**a**) ~25 µm and (**b**) ~1.5 µm.

Figure 9 shows the microstructure after micro-compression of 50% in different regions marked with small red squares as the EBSD observation areas in the top right of the picture. Figure 10 shows the contrast of the fraction of grain boundaries with misorientation angles in the center and edge regions of the compressed samples in pure Al with average grain sizes of ~25 µm and ~1.5 µm. Figure 9a,d shows the original microstructure before micro-compression tests of CG pure Al with an average grain size of ~25 µm and UFG pure Al with an average grain size of ~1.5 µm, respectively.

For the CG sample with an average grain size of ~25 µm, the microstructures in the center and in the edge area of the compressed samples after micro-compression of 50% are shown in Figure 9b,c, respectively. The results show that dislocation accumulation occurs and a lot of LAGBs are formed inside of the original large grains, both in the center and the edge region of the compressed samples, with average grain size of ~25 µm. Moreover, there are many recrystallized fine grains formed near the LAGBs in the center of the compressed sample, which is strikingly different compared to the microstructure in the edge region of the compressed sample, since the recrystallized grains are not found in this region. It is evident from the simulation results that the strain accumulation is higher as the position gets closer to the center of the compressed sample than that at the edge area [34]. Recrystallization easily occurs in the larger strain accumulation area and a lot of dynamic recrystallized fine grains can be observed in the center of the compressed sample, as shown in Figure 9b. The fractions of LAGBs and HAGBs have obvious changes, both in the center and the edge after micro-compression for the coarse-grained pure Al with an average grain size of ~25 µm, as shown in Figure 10a. The fraction of LAGBs in the center region of the compressed sample increases from 52.0% to 62.3% after micro-compression through a compression amount of 50%, which is smaller than that of 79% in the edge region of the compressed sample.

For the UFG sample with an average grain size of ~1.5 µm, the microstructures in the center and in the edge region of the compressed samples through a compression amount of 50% are shown in Figure 9e,f, respectively. The results demonstrate that the microstructures both in the center and edge regions of the compressed sample are maintained, with ultrafine grains with few sub-grain boundaries inside the small grains, similar to the original microstructure before micro-compression in Figure 9d. Meanwhile, there are few changes in the fractions of LAGBs and HAGBs for the UFG pure Al with an average grain size of ~1.5 µm before and after micro-compression, not only in the center, but also in the edge region of the compressed sample (Figure 10b). During micro-compression in UFG pure Al, dislocations cannot be restored and annihilated at grain boundaries. The mechanism is dominated by grain boundary sliding and rotation, which leads to little change in grain size and shape and misorientation angle distribution. There are some elongated grains in the edge region of the compressed samples, however, which is different compared to the microstructure in the center region as shown in Figure 9f. The formation of the elongated grains is related to the tensile stress in the edge region during micro-compression.

### 3.4. TEM Observation of Microstructure after Micro-Compression in Pure Al

**Figure 11:**
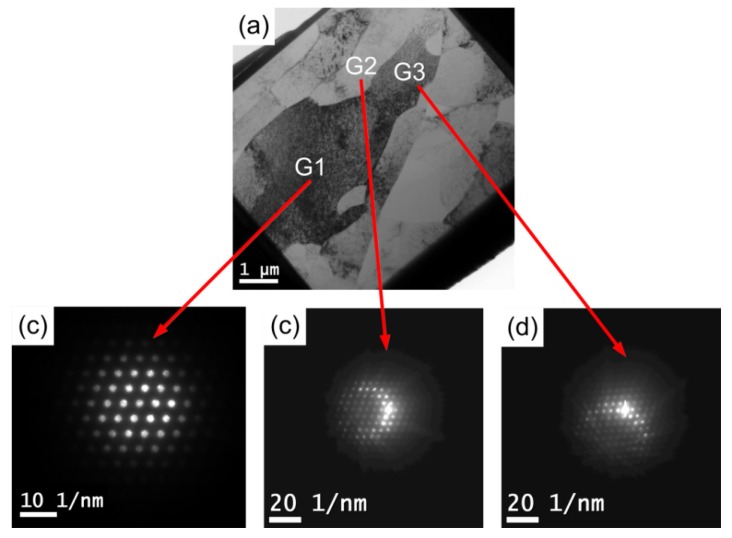
(**a**) Representative TEM microstructures at the center of compressed samples of the CG pure Al with an average grain size of ~25 µm: (**a**) bright field image and typical micro-diffraction patterns of the three neighboring grains marked with (**b**) G1, (**c**) G2 and (**d**) G3.

Figure 11 shows the representative TEM microstructures at the center of compressed samples of CG pure Al with an average grain size of ~25 μm. As seen in the bright field image in Figure 11a, the typical microstructure of CG pure Al sample subjected to compression is composed of coarse deformation bands. The micro-diffraction patterns of three neighboring grains G1, G2, and G3 were obtained as shown in Figure 11b–d. According to the three patterns, the misorientation angles between the three grains are estimated to be lower than 5°, which indicates that there are a lot of LAGBs formed in the coarse grains. Some small grains can also be found near the grain boundaries of the large grains. These results are consistent with the EBSD measurement results in Figure 9b, which also provide evidence of a grain interior deformation mechanism with dislocation sliding and dislocation accumulation during micro-compression of CG pure Al.

**Figure 12:**
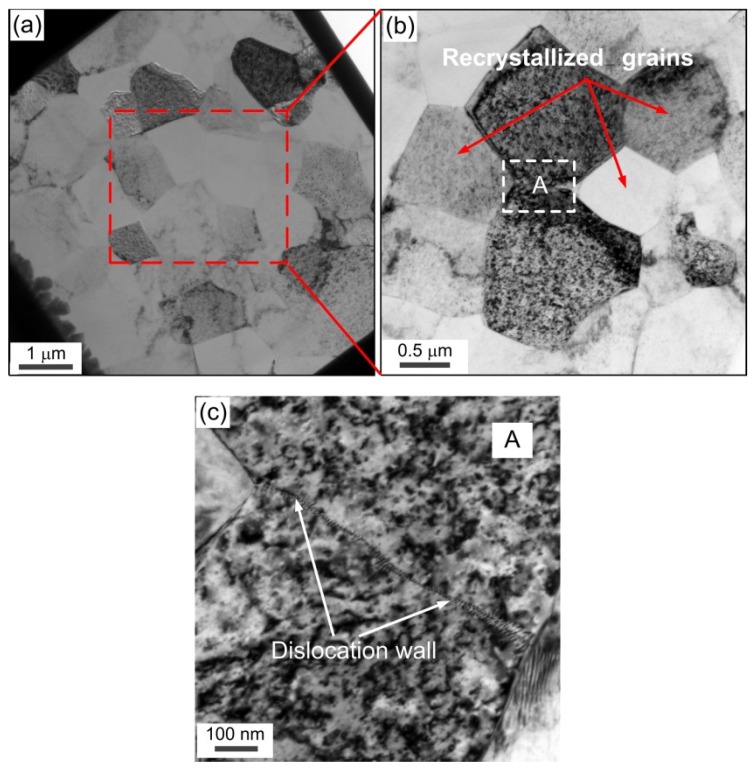
Representative TEM microstructures at the center of compressed samples of UFG pure Al with an average grain size of ~1.5 µm: (**a**) typical bright image; (**b**) magnified image of the dotted rectangle area in (**a**); (**c**) magnified image of the rectangle area in (**b**), showing subgrain boundary and dislocation wall.

Figure 12 shows the representative TEM microstructures at the center of compressed samples of the UFG pure Al with an average grain size of ~1.5 μm. Inspection of Figure 12a shows that ultrafine grains with an average grain size of ~1.2 μm were kept in the compressed sample of UFG pure Al, which is consistent with the result in Figure 9e. Some recrystallized grains without high density dislocations were observed in Figure 12b, which demonstrates that dynamic recrystallization occurred during compression. Additionally, small amounts of a subgrain boundary composed of a dislocation wall also existed in Figure 12c, which may be induced by dynamic recovery. This result also provides direct evidence that the generated dislocations are annihilated at the (sub) grain boundaries. In addition, the microstructural evolution after micro-compression of UFG pure Al demonstrates that the deformation mechanism at the micro/meso-scale is dominated by grain sliding and grain rotation in comparison with the TEM results of CG pure Al, shown in Figure 11. Meanwhile, the results indicate that the coordinated deformation ability can be improved significantly during micro-compression using UFG pure Al, and this shows that there is a strong potential for the use of UFG materials in micro/meso-forming technology.

## 4. Conclusions

(1) Micro-deformation behavior is transferred from work hardening to slight strain weakening with decreasing grain size, which is determined by the fraction of LAGBs and HAGBs formed during micro/meso-deformation. There is a corresponding transition from non-uniform deformation to uniform deformation after micro-compression testing with decreasing of grain size.

(2) A lot of LAGBs and recrystallized fine grains are formed inside of the original large grains in CG pure aluminum after micro-compression. In contrast, ultrafine grains are kept with few sub-grain boundaries inside the grains in UFG pure aluminum, which are similar to the original microstructure before micro-compression.

(3) The deformation mechanism at the micro/meso-scale in CG pure aluminum is dominated by grain interior deformation by shear (slip) bands, but for UFG pure aluminum, the deformation is changed with grain sliding and grain rotation with dynamic recrystallization and recovery.

(4) The surface roughness and coordinated deformation ability can be significantly improved during micro-compression with UFG pure aluminum, which demonstrates that the UFG materials have a potential application in micro/meso-forming.
